# Associations between Circulating Biomarkers of One-Carbon Metabolism and Mitochondrial D-Loop Region Methylation Levels

**DOI:** 10.3390/epigenomes8040038

**Published:** 2024-10-09

**Authors:** Andrea Stoccoro, Martina Lari, Lucia Migliore, Fabio Coppedè

**Affiliations:** 1 Department of Translational Research and of New Surgical & Medical Technologies, Medical School, University of Pisa, 56126 Pisa, Italy; andrea.stoccoro@unipi.it (A.S.); martina.lari@med.unipi.it (M.L.); lucia.migliore@unipi.it (L.M.); 2 Interdepartmental Research Center of Biology and Pathology of Aging, University of Pisa, 56126 Pisa, Italy

**Keywords:** DNA methylation, mitochondrial D-loop, one-carbon metabolism, vitamin B12, mitochondrial epigenetics

## Abstract

Background/Objectives: One-carbon metabolism is a critical pathway for epigenetic mechanisms. Circulating biomarkers of one-carbon metabolism have been associated with changes in nuclear DNA methylation levels in individuals affected by age-related diseases. More and more studies are showing that even mitochondrial DNA (mtDNA) could be methylated. In particular, methylation of the mitochondrial displacement (D-loop) region modulates the gene expression and replication of mtDNA and, when altered, can contribute to the development of human illnesses. However, no study until now has demonstrated an association between circulating biomarkers of one-carbon metabolism and D-loop methylation levels. Methods: In the study presented herein, we searched for associations between circulating one-carbon metabolism biomarkers, including folate, homocysteine, and vitamin B12, and the methylation levels of the D-loop region in DNA obtained from the peripheral blood of 94 elderly voluntary subjects. Results: We observed a positive correlation between D-loop methylation and vitamin B12 (r = 0.21; *p* = 0.03), while no significant correlation was observed with folate (r = 0.02; *p* = 0.80) or homocysteine levels (r = 0.02; *p* = 0.82). Moreover, D-loop methylation was increased in individuals with high vitamin B12 levels compared to those with normal vitamin B12 levels (*p* = 0.04). Conclusions: This is the first study suggesting an association between vitamin B12 circulating levels and mtDNA methylation in human subjects. Given the potential implications of altered one-carbon metabolism and mitochondrial epigenetics in human diseases, a deeper understanding of their interaction could inspire novel interventions with beneficial effects for human health.

## 1. Introduction

**Figure 1:**
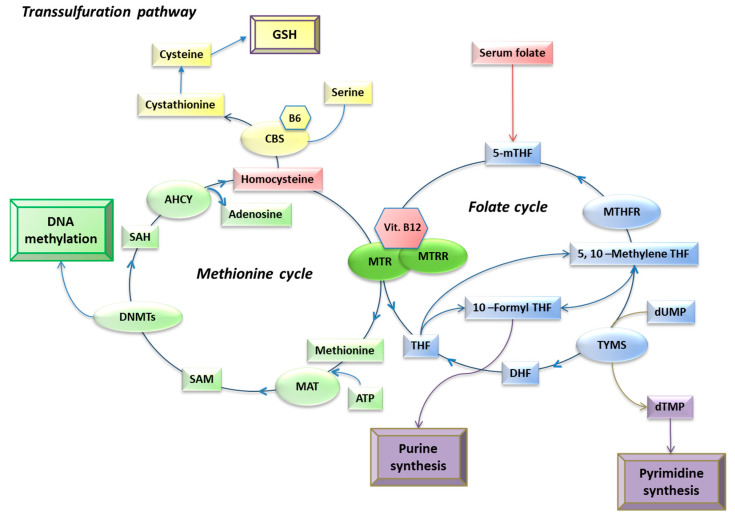
Schematic overview of one-carbon metabolism, adapted from [12]. Main metabolites (in rectangles), enzymes (in circles), and cofactors (in hexagons) are reported. In red are the one-carbon metabolism biomarkers analyzed in the current study. Abbreviations: 5-mTHF, 5-methyltetrahydrofolate; 5-10-methylene THF, 5,10-Methylenetetrahydrofolate; AHCY, S-Adenosylhomocysteine hydrolase; ATP, adenosine triphosphate; B6, vitamin B6; CBS, Cystathionine β-synthase; DNMTs, DNA methyltransferases; DHF, dihydrofolate; dUMP, deoxyuridine monophosphate; dTMP, deoxythymidine monophosphate; GSH, glutathione; MAT, methionine adenosyltransferase; MTHFR, methylenetetrahydrofolate reductase; MTR, methionine synthase; MTRR, methionine synthase reductase; TYMS, thymidilate synthase. Metabolites: 5-mTHF, 5-methyltetrahydrofolate; DHF, dihydrofolate; THF, tetrahydrofolate; dTMP, deoxythymidine monophosphate; dUMP, deoxyuridine monophosphate; SAH, S-adenosylhomocysteine; SAM, S-adenosylmethionine; Vit. B12, vitamin B12.

One-carbon metabolism is a critical pathway that utilizes folates for the synthesis of nucleotides, amino acids, antioxidant compounds, and methyl donor molecules [1]. Central to this pathway are the folate and methionine cycles (Figure 1). In the folate cycle, folates are reduced to 5-methyltetrahydrofolate (5-mTHF), which serves as the methyl donor for the remethylation of homocysteine (hcy) to methionine in the methionine cycle. The reaction is catalyzed by the enzyme methionine synthase (MTR), which transfers a methyl group from 5-mTHF to hcy, producing methionine and tetrahydrofolate (THF). The activity of MTR is supported by the cofactors vitamin B12 and the methionine synthase reductase (MTRR) enzyme [2]. Methionine is then converted to *S*-adenosyl methionine (SAM), the primary intracellular methyl donor, by methionine adenosyltransferase (MAT). SAM is predominantly used in methylation reactions, where it donates its methyl group to proteins and nucleic acids, subsequently being converted to S-adenosyl homocysteine (SAH) [3]. The SAH hydrolase (AHCY) then converts SAH into adenosine and hcy. Hcy can either re-enter the methionine cycle or be converted to cystathionine in the transsulfuration pathway, ultimately leading to the production of the antioxidant compound glutathione [4]. In the folate cycle, THF generated by MTR can be converted into 5,10-methylenetetrahydrofolate, which is used for nucleotide synthesis or to reconstitute 5-mTHF for hcy remethylation [5]. Circulating biomarkers of one-carbon metabolism, such as folate, hcy, and vitamin B12, are frequently assessed to evaluate the efficiency of this pathway, with altered levels being linked to various human diseases [6]. Plasma hcy levels are known to increase with age, leading to a hyperhomocysteinemia state, which is associated with geriatric conditions, including cardiovascular, neurodegenerative, and chronic kidney diseases [7]. Additionally, elevated hcy and reduced folate and vitamin B12 levels have been observed in the peripheral blood of patients with Parkinson’s disease (PD) [8], mild cognitive impairment (MCI), and Alzheimer’s disease (AD) [9] and individuals who have experienced an ischemic stroke [10]. Conversely, high circulating levels of folate and vitamin B12, along with low levels of hcy, have been found to be inversely correlated with the incidence of metabolic syndrome [11].

One of the most studied mechanisms regulated by one-carbon metabolism is DNA methylation. DNA methylation is an epigenetic modification involved in several cellular pathways, including genomic imprinting, X-chromosome inactivation, and regulation of gene expression. DNA methylation is catalyzed by DNA methyltransferases (DNMTs), specifically DNMT1, which is responsible for the maintenance of DNA methylation, and DNMT3A and DNMT3B, which are involved in de novo DNA methylation [13]. These enzymes use SAM as the methyl donor [14]. DNMTs typically methylate cytosine residues followed by guanine residues (CpG sites), but they can also methylate non-CpG sites [14]. Maintaining a proper balance in one-carbon metabolism is essential for physiological pathways regulated by DNA methylation. Indeed, the SAM:SAH ratio, which depends largely on the levels of one-carbon metabolites, reflects cellular methylation capacity, and its alteration could impair DNA methylation reactions [14]. Waterland and Jirtle were among the first to demonstrate that the supplementation of the diet with methyl donor groups, such as folic acid and vitamin B12, could alter mice phenotype through epigenetic mechanisms. Specifically, they observed a shift in color from yellow to brown, which was attributed to increased DNA methylation at critical CpG sites [15]. This groundbreaking study showed that mice with the same genotype can express different phenotypes through modifications in CpG methylation resulting from a diet rich in methyl donors. Further research in cell cultures and animal models demonstrated that deprivation of folate and vitamin B12 led to the upregulation of genes associated with AD, including *PSEN1* and *BACE1*, through the demethylation of their promoters [16,17,18,19]. This epigenetic alteration increased amyloid-beta production and contributed to disease neuropathology. Notably, these changes in DNA methylation and associated neuropathology were reversed when animals were fed with SAM supplements [16,18,19]. Similar associations were also reported in a human study, which identified a relationship among low folate, low vitamin B12, high hcy circulating levels, and low *PSEN1*, *BACE1*, *DNMT1*, *DNMT3A*, and *DNMT3B* gene methylation levels in blood DNA samples from individuals with AD [20]. Additionally, data from human intervention studies with folic acid or vitamin B12 showed that global DNA methylation levels increased following supplementation with both folic acid and vitamin B12 [21]. These findings from in vitro, in vivo, and human studies highlight a strong connection between nuclear DNA methylation and one-carbon metabolites. This relationship is particularly relevant to human conditions characterized by metabolic alterations and age-related diseases, where changes in metabolism significantly impact the epigenome, subsequently inducing changes in genomic functionality as an adaptive mechanism to restore metabolic homeostasis [22].

In addition to nuclear DNA, mitochondrial DNA (mtDNA) may also be regulated by epigenetic mechanisms, particularly through DNA methylation. Although the detection of methylated cytosine residues in mtDNA was reported over forty years ago, interest in mitochondrial epigenetics was renewed in around 2010 when studies began reporting mtDNA methylation in various tissues of animal and human origin [23,24]. These studies suggested a functional role for mtDNA methylation in the modulation of mtDNA replication and expression of mtDNA-encoded genes. Notably, the displacement loop (D-loop) sequence, the non-coding region of mtDNA, appears particularly sensitive to DNA methylation modifications. It can be bound by DNMTs and regulates the activity of mitochondrial transcription factor A (TFAM), a regulator of mtDNA transcription and replication [25,26]. Furthermore, DNMT1, DNMT3A, and DNMT3B have been identified inside mitochondria, indicating that the same enzymes involved in nuclear DNA methylation also participate in mtDNA methylation [24,27,28,29]. These DNMTs establish the mtDNA methylation pattern, which in turn regulates mitochondrial function and dynamics by modulating mtDNA transcription, already during embryogenesis [30,31,32]. Beyond regulating mtDNA expression and replication, de novo mtDNA methylation during early development protects mtDNA against oxidative damage, thereby contributing to the maintenance of mitochondrial homeostasis during the peri-implantation stage, an essential process for normal embryogenesis [33]. Interestingly, evidence suggests that prenatal exposure to various environmental factors can induce changes in mtDNA methylation and mitochondrial DNA copy number in newborns’ tissues, potentially impacting their health [30,34,35]. Additionally, associations have been noted between mtDNA methylation changes induced by particulate matter and welding fumes in exposed workers and an increased risk of cardiovascular disease [36,37,38]. Beyond embryonic development, mtDNA methylation is implicated in the processes of senescence and aging, likely reflecting the accumulation of mitochondrial damage. Indeed, mitochondrial activity declines with aging, and mtDNA methylation has been suggested to be sensitive to such decline [39]. By analyzing brain samples of 4- and 24-month-old mice, Dzitoyeva and coworkers observed that during aging, mtDNA 5-hydroxymethylcytosine levels decreased in the frontal cortex along with increased expression of mtDNA-encoded genes [39]. The authors suggested that mtDNA hydroxymethylation changes could be the consequence of the aging-induced impaired activity of epigenetic enzymes, including DNMT1. In line with this, it has been reported that mtDNA methylation could drive senescence, which contributes to the aging process. For example, methylation levels of the D-loop region and mitochondrial gene *MT-CO1* were found to be decreased in senescent cells compared to proliferative cells [40,41]. Moreover, the senescence process was associated with increased *MT-CO2* gene methylation levels, along with decreased protein expression [42]. Alterations in the methylation levels of D-loop, *MT-CO1*, and *MT-CO2* could greatly impact mitochondrial activity, thus contributing to the mitochondrial impairment that characterizes aging [25,26]. Thus, these studies strongly support the role of mtDNA methylation in age-related diseases [43]. Altered mtDNA methylation patterns have been observed in cellular models of various cancers, including colorectal cancer, glioblastoma, and osteosarcoma [44,45,46]. Of note, several studies have reported changes in mtDNA methylation levels, particularly in the D-loop region, in peripheral blood and central nervous system samples from individuals with neurodegenerative conditions such as MCI, AD, PD, and amyotrophic lateral sclerosis (ALS) [29,47,48,49]. Interestingly, D-loop methylation levels have been found to correlate with age and be sensitive to the progression of the neurodegenerative process [47,49]. Given the potential role of mtDNA methylation in human health, particularly in age-related disorders, a deeper understanding of the cellular mechanisms underlying its regulation is warranted. Since one-carbon metabolism is a well-known modulator of nuclear DNA methylation, it is plausible that it also influences mtDNA methylation. Some in vivo and in vitro studies suggest that betaine, a methyl donor of one-carbon metabolism, and hcy may modulate mtDNA methylation [50,51,52,53]. However, to the best of our knowledge, no studies until now have searched for associations between the circulating levels of one-carbon metabolites and mtDNA methylation levels in human subjects.

The current study aims to investigate whether circulating biomarkers of one-carbon metabolism, including folate, hcy, and vitamin B12, are linked to D-loop methylation levels in a cohort of neurologically healthy elderly individuals.

## 2. Results

### 2.1. Description of Sample Population

In this study, we analyzed blood samples from 94 elderly voluntary participants, for whom data on circulating levels of folate, hcy, and vitamin B12 levels were available. Serum folate levels ranged from 2.5 to 29.5 ng/mL, plasma hcy levels ranged from 4.1 to 34.2 µmol/L, and serum vitamin B12 levels ranged from 398 to 1200 pg/mL. D-loop methylation analyses were assessed in all subjects using the methylation-sensitive high-resolution melting (MS-HRM) technique, with methylation levels ranging from 0% to 11.1%.

### 2.2. Influence of Sex and Age at Sampling on mtDNA Methylation and Circulating Biomarkers of One-Carbon Metabolism

We first checked whether age and sex were associated with D-loop methylation status, folate, hcy, and vitamin B12 circulating levels.

**Figure 2:**
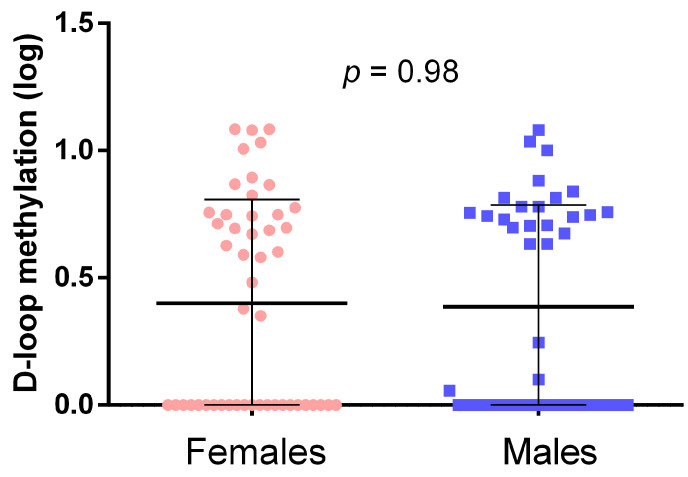
Mitochondrial D-loop methylation levels in females and males. The *p*-value was obtained with the Student-*t* test.

As shown in Figure 2, males and females exhibited similar D-loop methylation levels, with no significant difference in the mean D-loop methylation levels between the two groups.

**Figure 3:**
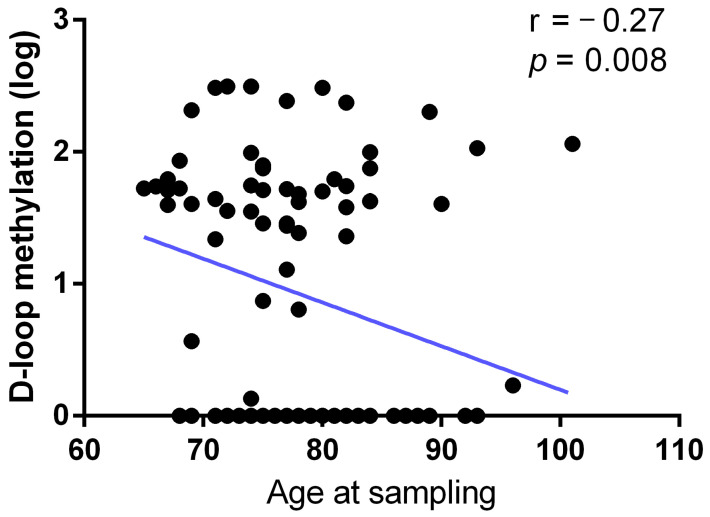
Correlation between D-loop methylation levels and age at sampling. The correlation was analyzed using Pearson’s correlation coefficient.

Moreover, the age at sampling of the subjects showed a statistically significant (r = −0.27; *p* = 0.008) inverse correlation with the D-loop methylation pattern (Figure 3).

**Figure 4:**
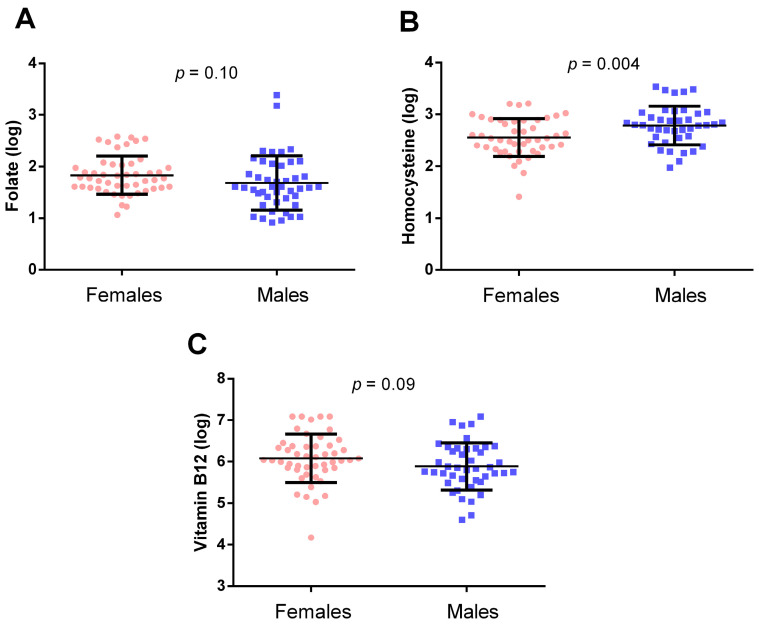
Folate (**A**), homocysteine (**B**), and vitamin B12 (**C**) in females and males. The *p*-value was obtained with the Student-*t* test.

Regarding one-carbon metabolism biomarkers, folate (Figure 4A) and vitamin B12 (Figure 4C) circulating levels did not significantly differ between males and females (*p* = 0.10 and *p* = 0.09, respectively). On the other hand, hcy levels (Figure 4B) were significantly lower in females compared to males (*p* = 0.004).

**Figure 5:**
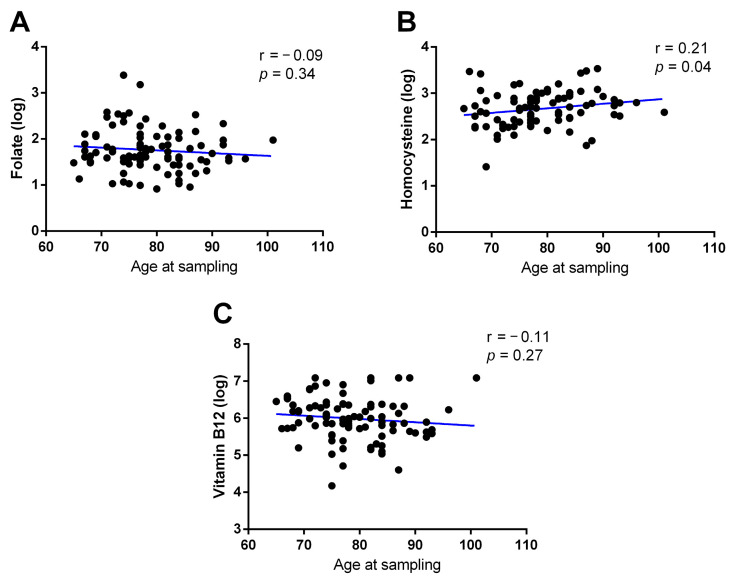
Correlation between folate (**A**), homocysteine (**B**), and vitamin B12 (**C**) circulating levels and age at sampling. The correlations were analyzed using Pearson’s correlation coefficient.

As shown in Figure 5, there was no correlation between age at sampling and folate levels (r = −0.09, *p* = 0.34; Figure 5A) or vitamin B12 (r = −0.11; *p* = 0.27; Figure 5C). On the other hand, a positive correlation was observed between age at sampling and hcy levels (r = 0.21; *p* = 0.04; Figure 5B).

### 2.3. Associations between Circulating Biomarkers of One-Carbon Metabolism and D-Loop Methylation

**Figure 6:**
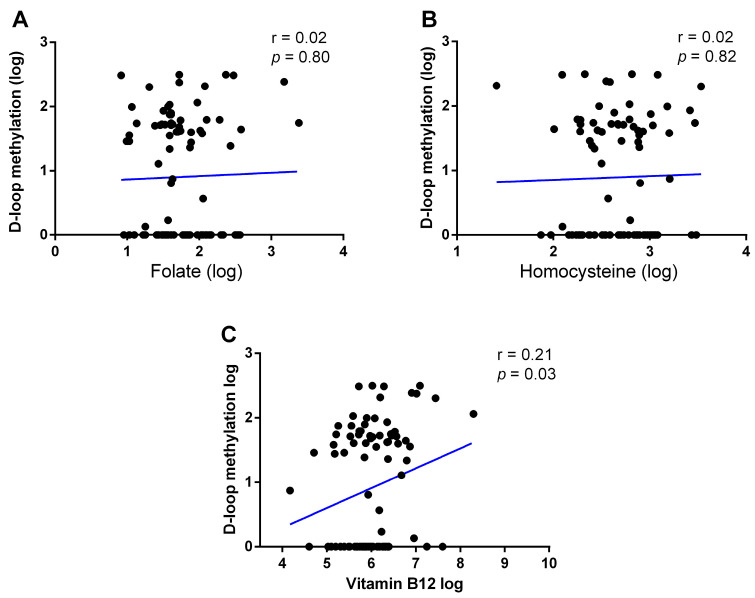
Correlations among D-loop methylation and folate (**A**), homocysteine (**B**), and vitamin B12 (**C**) circulating levels. The correlation was analyzed using Pearson’s correlation coefficient.

We then searched for correlations between peripheral blood D-loop methylation levels and one-carbon cycle metabolites (Figure 6). A significant positive correlation was found between D-loop methylation and serum vitamin B12 levels (r = 0.21; *p* = 0.03; Figure 6C). Otherwise, no correlation was observed between D-loop methylation and serum folate levels (r = 0.02; *p* = 0.80; Figure 6A), nor between D-loop methylation and plasma hcy levels (r = 0.02; *p* = 0.82; Figure 6B).

To further explore the correlation between D-loop methylation levels and circulating one-carbon metabolites, we subdivided the individuals based on the reference values provided by the center that conducted the biochemical analyses. The reference ranges for folate, hcy, and vitamin B12 were 4.6–18.7 ng/mL, 4.3–11.1 µmol/L, and 191–663 pg/mL, respectively. For folate, 25 individuals had levels below the reference range and 2 had levels above it. For hcy, 1 individual was below the reference range and 63 had levels above it. For vitamin B12, 10 individuals had levels below the reference range and 15 had levels above it.

**Figure 7:**
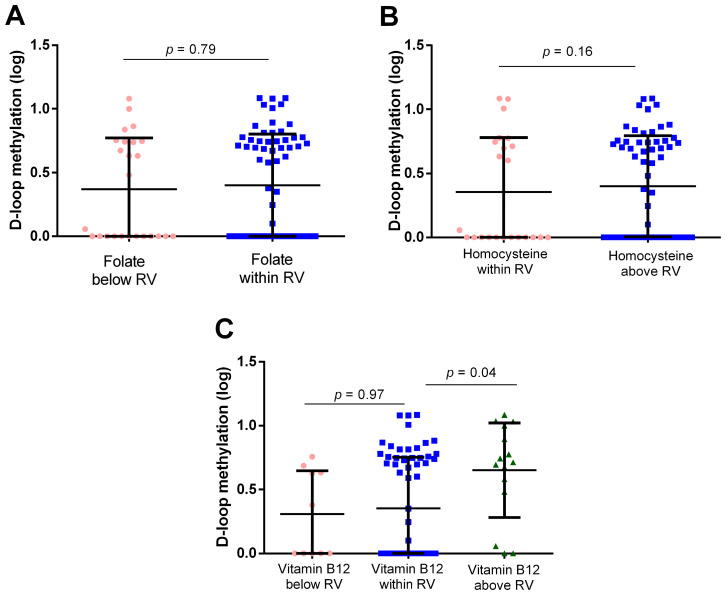
D-loop methylation levels in individuals below and within reference values (RV) of folate (**A**), within and above the RV of homocysteine (**B**), and in individuals below, within, and above the RV of vitamin B12 (**C**). *p*-value obtained with ANCOVA including age as the covariate.

D-loop methylation levels for individuals grouped according to these reference values are presented in Figure 7. Due to the limited number of individuals with serum folate levels above the range (*n* = 2) and those with hcy levels below the range (*n* = 1), comparisons were made between low and normal folate levels and between normal and high hcy levels. In contrast, there was a sufficient number of individuals with low, normal, and high vitamin B12 to allow comparisons among all three groups.

No significant differences in D-loop methylation levels were observed between groups for folate (*p* = 0.79; Figure 7A) and homocysteine (*p* = 0.16; Figure 7B). On the other hand, for vitamin B12, individuals with levels above the reference range showed higher D-loop methylation levels compared to those with levels within and below the reference range (Figure 7C). The difference was statistically significant, after Bonferroni correction, when comparing individuals with vitamin B12 above the reference range to those with levels within the reference range (*p* = 0.04). Conversely, no significant difference was observed between individuals with low and normal vitamin B12 values (*p* = 0.97).

**Figure 8:**
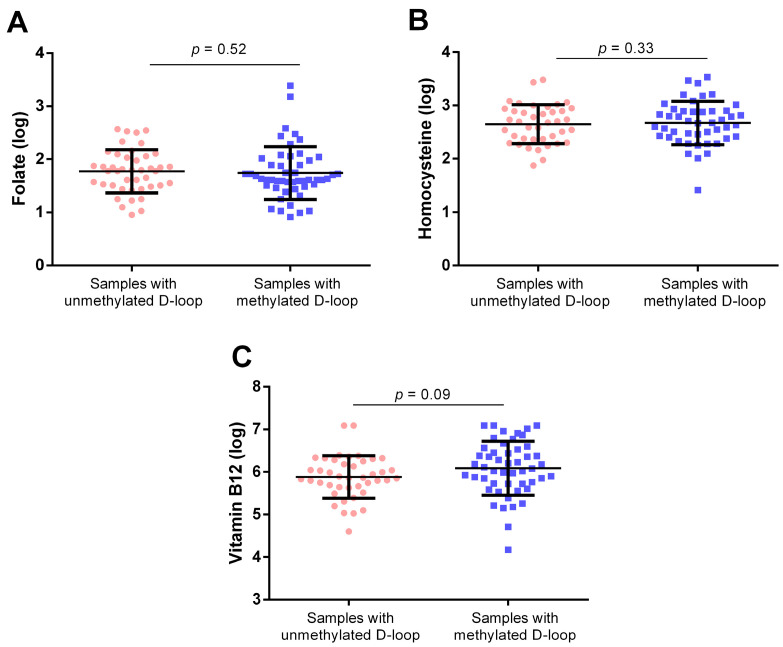
Comparison of folate (**A**), homocysteine (**B**), and vitamin B12 (**C**) circulating levels between samples with an unmethylated D-loop and those with a methylated D-loop. *p*-value obtained with ANCOVA including age and sex as covariates.

To further investigate the relationship between one-carbon metabolism biomarkers and D-loop region methylation, we compared samples with 0% methylation (*n* = 43) to those with D-loop methylation levels above 0% (*n* = 51). Although circulating vitamin B12 levels were lower in individuals with an unmethylated D-loop region compared to those with a methylated D-loop (Figure 8C), the difference was not statistically significant (*p* = 0.09). Similarly, circulating levels of folate (Figure 8A) and hcy (Figure 8B) did not differ significantly between individuals with unmethylated and methylated D-loop regions (*p* = 0.52 and *p* = 0.33, respectively).

## 3. Discussion

In the current study, we investigated the associations between circulating biomarkers of one-carbon metabolism, specifically folate, hcy, and vitamin B12, and the methylation pattern of the mitochondrial D-loop sequence in a group of elderly voluntary subjects. Our findings revealed a positive correlation between vitamin B12 and D-loop methylation, while no significant association was found between D-loop methylation and folate or hcy levels. Additionally, individuals with high vitamin B12 levels exhibited higher D-loop methylation compared to those with serum B12 levels within the normal range. Moreover, when comparing the distribution of folate, hcy, and vitamin B12 between individuals with unmethylated and methylated D-loop regions, we found that circulating vitamin B12 levels were higher in those with a methylated D-loop region, although the difference was not statistically significant. Age at sampling showed a positive correlation with both D-loop methylation and hcy levels. Moreover, males showed increased hcy levels compared to females.

Vitamin B12 plays a crucial role in cellular methylation reactions due to its involvement in hcy metabolism. Specifically, it serves as a cofactor for the methionine synthase enzyme, which catalyzes the transfer of a methyl group from 5-mTHF to hcy, forming methionine. Methionine is subsequently converted into SAM, the key molecule required for DNA methylation (Figure 1). Therefore, vitamin B12 is one of the essential molecules for DNA methylation processes. Researchers have explored whether DNA methylation is sensitive to vitamin B12 levels. Consistent with this, studies have associated nuclear DNA methylation with vitamin B12 serum levels, although with mixed results. For example, a negative correlation between global DNA methylation and vitamin B12 levels was found in an elderly population of healthy subjects and AD patients [54]. Similarly, in patients with colorectal cancer, serum vitamin B12 was inversely correlated with the overall DNA content of methylated 5-mC in both tumor tissue and peripheral blood [55]. However, a study involving ischemic stroke and healthy subjects showed a positive correlation between vitamin B12 and *MTHFR* gene methylation [56]. In contrast, no significant correlation was found between vitamin B12 serum levels and *MTHFR* gene methylation in a cohort of AD patients and healthy controls, while inverse and positive correlations were observed between *MTHFR* gene methylation and circulating hcy and folate levels, respectively [57]. Using artificial neural networks, we observed that AD patients are characterized by associations among low folate, low vitamin B12, and high hcy levels and decreased methylation of genes involved in AD, including *PSEN1* and *BACE1*, as well as genes encoding DNMTs, including *DNMT1*, *DNMT3A*, and *DNMT3B* [20]. Furthermore, a positive correlation between vitamin B12 and DNA methylation was observed at two CpG sites of the *IGFBP3* gene in cord blood [58] and also the *CYB27B1* gene in the peripheral blood of healthy elderly subjects [59]. However, some studies have found no significant correlations between circulating vitamin B12 levels and DNA methylation status. For instance, no association was observed between the overall content of methylated CpG sites in peripheral blood and circulating vitamin B12 levels in healthy volunteers [60,61,62] and in patients with neurodegenerative diseases [63]. Moreover, no association between vitamin B12 levels and *TERT* gene methylation was observed in subjects with essential hypertension [64]. Overall, there are indications that circulating vitamin B12 levels are associated with the methylation status of some nuclear loci, either positively or negatively.

The current study is the first to demonstrate a correlation between mtDNA methylation and circulating vitamin B12 levels. We observed a positive correlation between D-loop methylation and circulating vitamin B12 levels, with individuals exhibiting high vitamin B12 serum levels showing increased D-loop methylation compared to those with normal levels. Moreover, individuals with an unmethylated D-loop showed lower vitamin B12 levels compared to individuals with a methylated D-loop, although the difference was not statistically significant. The mitochondrial D-loop region is the regulatory sequence of mtDNA that modulates gene expression and replication [65]. Notably, decreased methylation of the D-loop has been associated with increased mtDNA copy number in cell cultures [66], human tumor tissues [44], and human peripheral blood [67]. Moreover, variations in D-loop methylation levels have been linked to the expression of mtDNA-encoded genes in cell cultures and human tissues [68,69], suggesting that methylation of this region significantly modulates mtDNA function. In line with this, an in vitro study showed that D-loop methylation regulated the activity of TFAM [25]. Additionally, several studies have reported the presence of DNMT1, DNMT3A, and DNMT3B inside mitochondria [27,28,29,70,71]. Furthermore, like nuclear DNA methylation, mtDNA methylation relies on SAM availability, which is imported into mitochondria by the mitochondrial SAM carrier encoded by the *SLC25A26* gene [72]. Thus, similar to nuclear DNA methylation, mtDNA methylation is closely linked to the proper function of one-carbon metabolism. Recently, it has also been proposed that mtDNA methylation may serve as a protective mechanism against mtDNA oxidation [33]. Impaired mtDNA methylation has been observed in various conditions characterized by increased oxidative stress and/or impaired one-carbon metabolism, such as blood DNA samples from ALS individuals with *SOD1* gene mutations [67], the postmortem brains of AD and PD patients [47], animal models of AD and ALS [29,47,73], and blood DNA samples from patients with MCI and AD [49], aging [39,42], cancer [44,45], and metabolic disorders [74,75], among others.

Although this is the first study to reveal a potential association between circulating levels of one-carbon metabolism biomarkers and mtDNA methylation, previous research has suggested a link between one-carbon metabolism and mitochondrial epigenetics. For instance, the muscle of piglets born from mothers fed a diet supplemented with betaine throughout gestation showed reduced D-loop methylation levels and increased expression of mtDNA-encoded genes, including *COX1*, *COX2*, and *ND5* [50]. Similarly, D-loop hypermethylation induced by the corticosterone treatment of fertilized eggs was counteracted by the injection of eggs with betaine [51]. Another study found that gilt polycystic ovaries were associated with a high homocysteine concentration in follicular fluid and the up-regulation of betaine homocysteine methyltransferase and glycine N-methyltransferase enzymes, both involved in one-carbon metabolism. Additionally, DNMT1 was upregulated in mitochondria, accompanied by increased methylation of *MT-RNR1*, *MT-RNR2*, and *MT*-*ND4* genes and the D-loop region [52]. They also observed that oocyte mitochondrial dysfunction induced by treatment with homocysteine and characterized by a reduced mtDNA copy number and gene expression and increased *MT-RNR2* methylation was reversed by co-treatment with the demethylating agent 5-Azacytidine, through the reduction in *MT-RNR2* gene methylation [53]. We observed that variants in genes involved in one-carbon metabolism and DNA methylation reactions were associated with D-loop methylation levels in a sample population including control subjects and AD patients [76]. Specifically, heterozygous *MTRR* 66AG carriers had higher D-loop methylation levels compared to wild-type *MTRR* 66AA individuals, and carriers of the rare AA genotype of *DNMT3A*-448A > G polymorphism had higher D-loop methylation levels than GG and GA genotypes carriers. Of note, the *MTRR* gene encodes for the 5-methyltetrahydrofolate-homocysteine methyltransferase reductase, which forms a complex with MTR for hcy remethylation to methionine in a reaction requiring vitamin B12 as a cofactor, further supporting the link between vitamin B12 and mtDNA methylation. In the current study, we did not observe any association between D-loop methylation and folate or hcy, suggesting that these one-carbon biomarkers do not influence the methylation of the mtDNA region analyzed. Further studies examining additional mtDNA regions are needed to clarify whether circulating folate and hcy can affect mtDNA methylation.

Additionally, the current study found an inverse relationship between D-loop methylation and age at sampling. This finding agrees with our previous reports where age was negatively correlated with the D-loop methylation of control subjects, individuals with PD, and in a cohort including control subjects and patients with dementia at different stages [48,49,77]. Similarly, an inverse correlation between the peripheral blood methylation of two cytosine residues in the *MT-RNR1* gene and age was observed in a cohort of individuals aged 18 to 91 years [78]. However, another study found that methylation of different CpGs in the *MT-RNR1* gene is positively correlated with age [79], suggesting that methylation of distinct mtDNA CpG sites may have different associations with age. For example, a study conducted on post-mortem brain tissue proposed that mtDNA methylation could be used to estimate chronological age [80]. The authors evaluated mtDNA methylation at both CpG and non-CpG sites in the nucleus accumbens and prefrontal cortex of drug users and non-users, creating an epigenetic clock based on mtDNA methylation data, and found accelerated mtDNA methylation age in drug users compared to non-users [80]. These findings indicate that mtDNA methylation may contribute to the aging process in humans.

Overall, the results of the current study support previous evidence of a potential link between one-carbon metabolism and mtDNA methylation [50,51,52,53,76]. Given the significant implications of mitochondrial DNA methylation for human health and disease [43], a deeper understanding of the factors involved in its establishment, maintenance, and modulation could offer valuable insights with potential clinical applications. If future studies confirm that D-loop methylation is modulated by vitamin B12, interventions targeting circulating vitamin B12 levels could be considered for individuals with diseases characterized by altered mtDNA methylation. Evidence suggests that changes in dietary intake of folate and vitamin B12 could ameliorate the aging process by modifying age-associated DNA methylation changes and subsequently altering age-associated physiologic and pathologic processes [14]. It is plausible that similar effects could occur with mitochondrial DNA methylation. Of note, there is a significant interplay between one-carbon metabolism, mitochondria, and the epigenetic regulation of both nuclear and mitochondrial DNA. One-carbon metabolism occurs across the cytoplasm, nucleus, and mitochondria, with enzymes and substrates synthesized in the three cellular compartments. Mitochondria provide essential metabolites for one-carbon metabolism, such as ATP, α-ketoglutarate, β-nicotinamide adenine dinucleotide, and acetyl coenzyme A, which are crucial for DNA and histone tails’ epigenetic modification [81]. Additionally, mtDNA genetic variants and copy number influence nuclear DNA methylation patterns at specific loci [82,83]. Thus, mitochondria play a pivotal role in modulating nuclear epigenetic mechanisms. Conversely, nuclear epigenetic modifications regulate the expression of genes encoding proteins that regulate mtDNA gene expression and mtDNA replication [84,85]. This emerging evidence highlights the complex epigenetic crosstalk between the nucleus and mitochondria, which is regulated by one-carbon metabolism. This metabolism, in turn, is influenced by enzymes and cofactors critical for the proper gene expression of both nuclear and mitochondrial DNA. Thus, understanding the mtDNA methylation effect on one-carbon metabolism could aid in the design of new treatment approaches for diseases characterized by impairment in one-carbon metabolism. For example, since AD is characterized by altered levels of folate, hcy, and vitamin B12 as a consequence of mitochondrial dysfunction that reduces the availability of important one-carbon metabolism co-factors, dietary interventions aimed at enhancing mitochondrial activity have been proposed [86]. The findings of the current study, which detected the link between one-carbon metabolism and mtDNA methylation in the peripheral blood of elderly subjects, provide a foundation for future research aimed at elucidating the pathological mechanisms underlying diseases associated with impaired one-carbon metabolism.

We acknowledge the limitations of the current study, which was conducted on a relatively small cohort of neurologically healthy individuals. While our findings suggest a potential link between mtDNA methylation and one-carbon metabolism, these results need to be validated in larger cohorts. This should be considered as a preliminary study that could inspire novel investigations aimed at examining the associations between one-carbon metabolism and mitochondrial epigenetics. Furthermore, as the present data pertain to elderly subjects, additional studies involving younger individuals are necessary to determine if the observed associations change with age. It is also important to note that the participants in this investigation were initially enrolled as a control group in a study aimed at identifying epigenetic biomarkers for Alzheimer’s disease [57]. While these individuals were neurologically healthy and deemed healthy overall, we cannot entirely rule out the presence of other age-related chronic conditions that could impact one-carbon metabolism. Moreover, we employed a technique, the MS-HRM, that does not allow us to obtain information on the methylation levels of specific CpG sites, providing only the average methylation levels of the 10 CpG sites included in the D-loop amplicon analyzed. We observed a relatively high frequency of individuals with an unmethylated D-loop, although we cannot exclude that some of the 10 CpG sites could show some methylation degree above 0%. However, we have previously observed that D-loop methylation levels measured using the MS-HRM are highly comparable to those obtained using pyrosequencing, the gold standard for gene-specific methylation analysis that provides information on the methylation levels of specific CpG sites. Future studies focusing on methylation levels at individual CpG sites may provide deeper insights into the relationship between circulating vitamin B12 and D-loop methylation levels. Nonetheless, the significant contribution of this study is that it is the first to explore the relationship between circulating one-carbon metabolism biomarkers and mtDNA methylation in humans. Further investigations in populations with impaired one-carbon metabolism, such as patients with AD, PD, or other age-related diseases, are needed to clarify whether and how impairments in one-carbon metabolism might lead to alterations in mtDNA methylation. Additionally, further investigations should also aim to investigate whether mtDNA methylation and gene expression levels can be modulated by molecules, such as SAM, that could potentially counteract the impairment of one-carbon metabolism.

## 4. Materials and Methods

### 4.1. Main Information on Enrolled Subjects in the Study

The current investigation involved 94 healthy volunteers from our previous study in which D-loop methylation was assessed in peripheral blood DNA samples and for whom data on circulating biomarkers of one-carbon metabolism were available [70]. The cohort included 49 females and 45 males, all of whom were Caucasians of Italian origin residing in Tuscany, with a mean age at sampling of 78.6 ± 7.5 (mean ± standard deviation). Participants were recruited at the Department of Neuroscience, University of Pisa (Pisa, Italy), as part of a study conducted at the Pisa University Hospital aimed at identifying epigenetic biomarkers for Alzheimer’s disease [57]. All individuals underwent clinical and neurological examinations at recruitment. Subjects prescribed medication or supplemented with vitamins that could influence DNA methylation were excluded from the study. All included individuals were found to be healthy and free from neurological complications [87]. No other anthropometric data, including blood pressure, BMI, or the presence or absence of other chronic conditions, were available. Written informed consent was obtained from all participants before their enrolment. The study was approved by the ethics committee of the Pisa University Hospital (protocol number: 3618/2012).

### 4.2. One-Carbon Metabolism Biomarker Analyses

Analyses of circulating levels of folate, homocysteine, and vitamin B12 were conducted at Pisa University Hospital, as previously described [88]. Briefly, following blood collection via peripheral venipuncture, serum and plasma were immediately separated and stored at −80 °C until assayed. Serum levels of folate and vitamin B12 were determined using an electrochemiluminescence immunoassay, while plasma homocysteine levels were measured via liquid chromatography–tandem mass spectrometry. Table 1 presents the observed concentration ranges of folate, hcy, and vitamin B12 in our samples, along with the reference ranges provided by the clinical analysis laboratory at Pisa Hospital that performed the analyses, and the distribution of individuals with values below, within, or above the reference ranges.

### 4.3. Methylation-Sensitive–High-Resolution Melting (MS-HRM) Analyses for the Quantification of mtDNA Methylation

Methylation analyses of the mitochondrial D-loop region were performed using the MS-HRM technique, as fully detailed elsewhere [70]. Briefly, DNA samples were extracted from whole peripheral blood collected in EDTA tubes using a QIAmp DNA blood Mini Kit (Qiagen, Milan, Italy, Catalog N 51106) and quantified using a NanoDrop ND 2000c spectrophotometer (NanoDrop Thermo scientific, Wilmington, DE, USA). Two hundred nanograms of each sample of the DNA were bisulfite-treated with an EpiTect Bisulfite Kit (Qiagen, Milan, Italy). MS-HRM analyses included a PCR amplification followed by an HRM step for melting analysis. The protocol of the PCR reactions was as follows: an initial step at 95 °C for twelve minutes, 50 cycles of 95 °C for thirty seconds, 56 °C for forty-five seconds, and 72 °C for forty-five seconds. Table 2 reports the main characteristics of the amplicon analyzed. The protocol of melting analysis was as follows: a denaturation step at 95 °C for 10 s followed by a renaturation step at 50 °C for 60 s and a rapid increase in temperature at 65 °C for 0.2 °C every 15 s to 95 °C. Samples were tested in duplicate in each MS-HRM reaction across at least two independent experiments. For each MS-HRM assay, standard curves were generated using DNA samples with known methylation status ranging from 0 to 100% methylation levels, prepared by mixing fully methylated and fully unmethylated DNA (Qiagen, Milan, Italy). Specific DNA methylation levels were determined using a MatLab (The MathWorks, Inc., Natick, MA, USA) interpolating function.

### 4.4. Statistical Analysis

Circulating folate, homocysteine, vitamin B12 levels, and mtDNA methylation data were tested for normality with the Shapiro–Wilk test. As all variables exhibited skewed distributions, logarithmic transformation of the data was applied before the statistical analyses. Correlations between one-carbon metabolism biomarker circulating levels, age at sampling, and D-loop methylation were evaluated using the Pearson correlation coefficient. Differences between females and males were assessed using Student’s *t*-test. To examine one-carbon metabolism biomarker circulating levels and D-loop methylation among groups, analysis of covariance (ANCOVA) with age and/or sex as the covariates was used, followed by post hoc Bonferroni correction for multiple comparisons. Statistical analyses were conducted using STATGRAPHICS 5.1 (Statgraphics, The Plains, VA, USA) and MedCalc (MedCalc Software, Ostend, Belgium). The statistical power of the study was calculated using G*Power, (version 3.1.9.7; Heinrich-Heine-Universität Düsseldorf, Düsseldorf, Germany). With the available sample size, we had an 80% statistical power to identify a correlation with a Pearson coefficient of 0.25 and a *p*-value of 0.05.

## 5. Conclusions

The current study showed for the first time that blood mitochondrial DNA methylation levels are associated with vitamin B12, a peripheral biomarker of one-carbon metabolism. Given the involvement of both mtDNA methylation and one-carbon metabolism in human health status, a better understanding of their interplay could shed new light on the etiological pathway underlying diseases characterized by impairment in one-carbon metabolism or altered mitochondrial epigenetic mechanisms. In this way, interventions aimed at modulating one-carbon metabolism with effects on mtDNA methylation or that induce changes in mtDNA epigenetics that enhance mitochondrial activity with improvement in one-carbon metabolism could have beneficial effects on affected individuals.

## Figures and Tables

**Table 1:** One-carbon metabolism biomarkers: circulating levels and sample distribution.

	Folate	Homocysteine	Vitamin B12
Sample concentration ^a^	6.5 ± 4.0 ng/mL (2.5–29.5)	15.4 ± 6.0 µmol/L (4.1–34.2)	469.5 ± 275.0 pg/mL (398–1200)
Reference concentration ^b^	4.6–18.7 ng/mL	4.3–11.1 µmol/L	191–663 pg/mL
Samples below the reference range ^c^	25 (26.6%)	1 (1.1%)	10 (10.6%)
Samples within the reference range ^c^	67 (71.3%)	30 (31.9%)	69 (73.4%)
Samples above the reference range ^c^	2 (2.1%)	63 (67.0)	15 (16%)

^a^ Data presented as the mean ± standard deviation (range); ^b^ reference ranges provided by the laboratory; ^c^ data presented as frequency (%).

**Table 2:** Primers’ main characteristics.

	Sequence (5′→3′)	Annealing Temperature	Amplicon Size	Nucleotide Position	Number of CpG Sites
**Primer forward**	GGAGTTTTTTATGTATTTGGTATTTT	56 °C	222 bp	35–256 (GenBank: J01415.2)	**10**
**Primer reverse**	ACAAACATTCAATTATTATTATTATATCCT				

## Data Availability

The data analyzed during the current study are available from the corresponding author upon reasonable request.
